# Astatine-211 based radionuclide therapy: Current clinical trial landscape

**DOI:** 10.3389/fmed.2022.1076210

**Published:** 2023-01-06

**Authors:** Per Albertsson, Tom Bäck, Karin Bergmark, Andreas Hallqvist, Mia Johansson, Emma Aneheim, Sture Lindegren, Chiara Timperanza, Knut Smerud, Stig Palm

**Affiliations:** ^1^Department of Oncology, Sahlgrenska University Hospital, Gothenburg, Sweden; ^2^Department of Oncology, Institute of Clinical Sciences, Sahlgrenska Academy, University of Gothenburg, Gothenburg, Sweden; ^3^Department of Radiation Physics, Institute of Clinical Sciences, Sahlgrenska Academy, University of Gothenburg, Gothenburg, Sweden; ^4^Smerud Medical Research International AS, Oslo, Norway

**Keywords:** targeted alpha therapy, alpha particle, astatine-211, radionuclide, human, clinical trial

## Abstract

Astatine-211 (^211^At) has physical properties that make it one of the top candidates for use as a radiation source for alpha particle-based radionuclide therapy, also referred to as targeted alpha therapy (TAT). Here, we summarize the main results of the completed clinical trials, further describe ongoing trials, and discuss future prospects.

## 1. Introduction

Astatine was first synthesized at the University of California, Berkley in 1940 (1), and the first report of its treatment on humans was published as early as 1954 (2). Because astatine lacks stable or long-lived isotopes, it is named after the ancient Greek word “astatos' meaning “unstable'. Astatine is often referred to as “the rarest element on earth” because only isotopes 214–219 can be found naturally in the earth's crust in equilibrium with uranium. It is estimated that there are only ~0.07 grams present at any given time. This makes availability an issue. However, substantial amounts of astatine-211 (^211^At) can be produced in cyclotrons. The availability, chemistry, and logistics of handling this rare element have been comprehensively addressed recently (3–6) and will be briefly mentioned here.

^211^At has a 100% alpha emission with only one alpha particle emitted per decay, which prevents unpredictable dose localization caused by the detachment of radioactive daughters from the carrier vector. This is comparable to other alpha emitters such as thorium-227 (^227^Th), radium-223 (^223^Ra), lead-212 (^212^Pb), bismuth-212 (^212^Bi), and actinium-225 (^225^Ac), all of which have a long decay series and may suffer from recoil problems. A half-life (t$\frac{1}{2}$) of 7.2 h is also another advantage, with <1% of radioactivity remaining after 2 days, which may decrease normal tissue exposure, while still being long enough to be shipped for up to 3 h to perform chemistry/radiopharmacy with enough remaining activity to use for clinical treatment. This review summarizes the current clinical experiences with ^211^At-based treatments, provides an update on ongoing trials, and provides perspectives on possible paths that may be explored in the near future.

## 2. The past

### 2.1. Summary of the main findings from completed clinical experiences with ^211^At

To the best of our knowledge, the first documented use of ^211^At in humans was published in 1954, when Hamilton et al. investigated its potential use in the treatment of thyroid disorders (2). Thereafter, a case report was published in 1990 where a patient with an inoperable carcinoma of the tongue received intra-arterially injected ^211^At-labeled human serum albumin microspheres as a palliative measure (7). A few conclusions can be drawn from these very early works in humans treated with ^211^At, namely, it can accumulate in the thyroid tissue, and alpha-emitting nuclides possess enormous destructive capacity when locally retained. Two published phase I trials used an intra-cavitary route of administration, whereby systemic exposure was minimized and no systemic toxicity could be detected (8, 9). Importantly, both studies calculated locally high absorbed doses in the treated volume that was beneficial to the patients. Signs of this were also found in both studies, with some patients surviving longer than expected, but with a clear risk of biased inclusion. These findings should be explored in correctly designed efficacy-seeking trials. Table 1 summarizes human experiences and the completed clinical trials performed so far.

**Table 1:** Completed clinical studies using ^211^At. (*NTC number*) is the ClinicalTrials.gov identifier.

**Institution, Reference**	**Clinical situation**	**Nb. Pts**.	**Study Objective**	**TAT-agent**	**Target**	**Adminis-tration**	**Act-ivity**	**Toxicity/effect**
Duke University Medical Center, Durham, USA (9) (*NCT00003461*)	Recurrent surgically resected glioblastoma	18	Feasibility and safety	^211^At-ch81C6	tenascin	Surgically created resection cavity	71–347 MBq	MTD, Not reached
Sahlgrenska University Hospital, Gothenburg, Sweden (8, 10–12) (*NCT04461457*)	Relapsed ovarian cancer	12	Safety, Toxicity Pharmacokinetics	^211^At-MX35 F(ab')2	NaPi2b	Intra peritoneal	34–355 MBq	MTD, Not reached
Carl Gustav Carus University Hospital, Dresden, East Germany (7)	Recurrent carcinoma of the tongue	1	Palliation	^211^At-labeled human serum albumin microspheres (15–25μm)	Tumor vasculature	Intra arterially (left lingual artery)	200 MBq	Tumor necrosis/ tongue necrosis
University of California Berkeley and San Francisco, USA (2)	Thyroid gland disorders	8	Tracer study	^211^At	Na^+^/I^−^ symporter (NIS)	Per oral in 25ml water	1.85 MBq	Thyroid uptake was established

#### 2.1.1. Berkeley, California, USA 1954

Knowing that the halogen iodine can accumulate in thyroid tissue, the same year that ^211^At was discovered in 1940, it was investigated for potential accumulation in thyroid tissues in guinea pigs. Due to other matters, research in this area was halted for several years. In 1954 at the Crocker Laboratory in Berkeley California, Hamilton et al. (2) investigated the thyroid accumulation of ^211^At in 7 patients with various thyroid disorders and one papillary adenocarcinoma with cervical lymph node metastases. Here, 1.85 MBq ^211^At was dissolved in 25 mL water and given orally to the patients 13–22 h prior to surgery to remove the thyroid gland. From this small data set, it was concluded that the accumulation of ^211^At in the thyroid glands was relatively higher than that observed in experiments using rats. Additionally, a correlation was observed between ^211^At uptake and stable iodine in the thyroid tissue. There was no discernible accumulation of ^211^At in the cervical lymph node metastases present in the patient with papillary adenocarcinoma. No toxicity or adverse events were reported.

#### 2.1.2. Dresden, East Germany 1990

In a case report by Doberenz et al. (7) at the Carl Gustav Carus University Hospital in Dresden, East Germany, a patient with an unresectable recurrent carcinoma of the tongue was treated in 1988 with 200 MBq of ^211^At-labeled human serum albumin microspheres that were 15–25 μm in diameter. The radio-conjugate was injected directly into the left lingual artery. Although the tumor tissue supplied by the artery successfully became necrotic within a few days, necrosis eventually spread to the entire tongue. Locally, in the tumor, the dose was calculated to be 302 Gy, and by day 30, no viable tissue was left in the tongue. At 4 and 20 h post injection, 81 and 64% of the radioactivity was found in the tongue, respectively. The thyroid gland was blocked for up-take. In the thyroid, <1 and 3% were found at 4 and 20 h respectively. A slight depression was found in thyroid hormone levels, but within normal range. Using a relative biological effectiveness (RBE) of 7, the lungs were calculated to receive 5.32 Sv. The patient died on day 43 of apparent aspiration pneumonia. The autopsy revealed no signs of pneumonitis in the lungs. Histological examination of the thyroid gland showed atrophy and fibrosis.

#### 2.1.3. ***Durham, North Carolina, USA, 2008***

In 2008, Zalutsky et al. (9) reported the first completed clinical trial using targeted alpha therapy with ^211^At at the Duke University Medical Center. ^211^At was conjugated to the chimeric (human/mouse) mAb, anti-tenascin, and ch81C6. Tenascin is an extracellular matrix glycoprotein ubiquitously expressed in high-grade gliomas, but not in normal brain tissue. This clinical trial was initiated following a series of well-performed and relevant preclinical investigations with the construct ^211^At-ch81C6, demonstrating *in vitro* cytotoxic effects (13), *in vivo* stability (14), tissue distribution after i.v. (intravenous) and intrathecal administration in mice for calculation of human radiation doses (15), and investigations on long-term toxicity and the maximum tolerated activity in mice (16). Considering that the t$\frac{1}{2}$ of ^211^At is 7.2 h, systemic exposure and product degradation could be minimized by choosing a local administration route. Therefore, ^211^At-ch81C6 was administered into a Rickham reservoir and its catheter was placed in the surgically created resection cavity.

This phase I dose escalation study (NCT00003461) was performed between April 1998 and June 2001 to study the feasibility and safety of locally injected ^211^At-ch81C6 into the resection cavity of recurrent brain cancer. This study followed as a natural extension after promising results were obtained using beta-particle-emitting constructs with murine-81C6 in similar clinical situations (17, 18). It was argued that the advantages of alpha particle vs. beta particle irradiation could prove to be maximized in this clinical setting, i.e., the risk of small pockets of remaining malignant cells with a low blood supply. Here, the alpha particle has the advantages of a lower sensitivity to tumor oxygenation and a higher RBE (relative biologic effect) owing to its high linear energy transfer (LET) and shorter path length, which would be beneficial and possibly less toxic.

Nineteen patients were enrolled, 18 of which (nine female) were treated for recurrent brain cancer (glioblastoma multiforme, n = 14; anaplastic oligodendroglioma, *n* = 3; anaplastic astrocytoma, *n* = 1). One patient was excluded because of subgaleal leakage seen in the postoperative flow study with technetium-99 m-labeled albumin. This was performed to verify the Rickham catheter patency, and to ensure that the resection cavity was not communicating with the subarachnoid space (i.e., intrathecal communication). Astatine is a halogen that shares several chemical properties with iodine, whereby uptake in tissues expressing the sodium/iodide symporter (NIS) can be significantly blocked with an excess of iodine. Therefore, all patients received blocking with daily administration of potassium iodine and liothyronine sodium (from 48 h. before initiation to 16 days after the therapy).

Activities of ^211^At ranged from 71 to 347 MBq and were conjugated to 10 mg ch81C6 and administered in <6 mL. Four activity levels were identified: 71–104 MBq (*n* = 5), 135–148 MBq (*n* = 7), 215–248 MBq (*n* = 5), and one patient received 347 MBq.

No dose-limiting toxicity was recorded, hence the maximum tolerated dose (MTD) was not identified. Grade 2 headache (*n* = 3), expressive aphasia (*n* = 1), hand numbness (*n* = 1), and quadrant anopsia (*n* = 1) were all possibly attributable to the treatment. All of these resolved within a few weeks, except for the visual deficit. No correlation with administered activity was found. The most common adverse reaction recorded during follow-up was seizures (two with grade 2, three with grade 3 and one with grade 4), but all these occurred during disease progression and therefore were not considered dose-limiting. There was one case of aplastic anemia that occurred 5 weeks after a single dose of chemotherapy (lomustine) was administered, due to recurrent disease 3 months after treatment with 74 MBq ^211^*At-ch81C6*. Furthermore, one patient developed a second malignancy, an undifferentiated anaplastic small-cell neoplasm with neuroblastic features in the neck, 8 weeks after treatment with 215 MBq of ^211^*At-ch81C6*. None of these events can be considered due to the treatment, but it is important to keep in account while more experience is gathered.

Serial gamma-camera imaging (of the very minute 77 to 92 keV polonium K X-rays emitted during ^211^At decay) demonstrated limited catabolism and excellent stability. It was calculated that 96.7% ± 3.6% of all decay occurred within the resection cavity, and correspondingly, the total activity in the blood pool was generally <0.5% ID (injected dose) at all time points up to 24 h. It was concluded that this therapy was feasible and could be delivered safely. Although there were few patients and a risk of biased inclusion, the median overall survival rate of 52 weeks for the glioblastoma patients treated was superior to the literature data.

#### 2.1.4. Gothenburg, Sweden, 2009

From February 2005 to March 2011, 12 patients with recurrent ovarian cancer were treated with ^211^*At-MX35-F(ab')*_2_, at the Sahlgrenska University Hospital in Gothenburg Sweden, as first reported by Anderson et al. (8) in 2009. MX35 is a murine IgG mAb targeting the NaPi2b (SLC34A2) cell surface glycoprotein, which is expressed in >90% of human epithelial ovarian cancers.

Radioimmunotherapy based on a beta-emitting radionuclide (yttrium-90) has previously failed to show an effect on overall survival (OS) in a phase 3 randomized trial aimed at preventing local relapse in small-scale disease ovarian carcinoma (19). As shown by biokinetic modeling (20), part of the failure could be because beta-emitting therapy does not reach a sufficiently high dose to eradicate single cells or small cell clusters, which is believed to be the reason for relapse. Numerous preclinical studies have demonstrated dramatic effects using alpha-particle-emitting radionuclides to treat small-scale diseases in mouse models with peritoneal growth of ovarian cancer (21–23). Organ tolerance for the kidney and peritoneal lining, as well as the RBE for bone marrow, were separately investigated in mice using the radiation-sensitive BALB/c strain (24–26).

The aim of this dose-escalation study (NCT04461457) was to investigate the safety and pharmacokinetics of ^211^*At-MX35 F(ab')*_2_ using a 3+3 design. To mimic the gross tumor-free adjuvant situation with an undisturbed peritoneal lining, only patients with recurring epithelial ovarian cancer treated with salvage chemotherapy to achieve complete or good partial remission were included. A total of 12 patients were treated with one intraperitoneal (i.p.) infusion of ^211^*At-MX35 F(ab')*_2_ in 1–2 L of Extraneal^®^ solution, which was evacuated after 24 h. The treatment was well-tolerated and escalated from 20 to 215 MBq L^−1^ without any dose-limiting toxicities. The most frequent toxicities were low grade, related to the catheter procedure, and generally resolved within a few days; one grade 4 toxicity was due to perforation of the small intestine after catheter insertion (10). No link was found between registered toxicity and radiation exposure. Some patients experience fatigue and nausea, which are known to be frequent radiation-induced side effects. However, these side effects could also be explained by the procedure due to frequent around-the-clock blood sampling, imaging, and the extended abdomen, making low-grade insomnia frequent. No late toxicities were found for thyroid, renal, or bone marrow function. One patient had a new malignancy 2.7 years after treatment, which was later diagnosed as Lynch syndrome (10). Pharmacokinetics with corresponding calculations of normal tissue dose showed low doses (11), which corresponds well with the absence of hematological and biochemical changes (8, 10). By not using thyroid blocking agents in the lowest activity cohort, the thyroid uptake of free ^211^At and estimation of the effect of blocking could be performed (8). The following patients received potassium perchlorate 200 mg twice daily from day−1 to day 2.

The absorbed doses for this treatment were calculated (11) and amounted to >2 Sv for 200 MBq L^−1^. However, the term effective dose should not be used for any radiotherapy as stated by the International Commission on Radiological Protection (ICRP) (27) and, for alpha-particle irradiation, a conservative radiation weighting factor of 20 is applied, whereby the risk might be overestimated. To circumvent the problem of an unknown weighting factor, another epidemiologically based approach was used (12). Here, organ-dose data from the same phase I study were used together with published data on cancer development following exposure to alpha-particle-containing medication. Using this epidemiologically based method, the risk of secondary cancers following i.p. therapy with ^211^At-mAb was estimated. The resulting estimates varied from 0.11 to 1.84 excess cases per 100 treated (by the i.p. route with 200 MBq L^−1^ of ^211^At-mAb), depending on the use of various assumptions made (e.g., age at treatment, low-LET equals high-LET, or competing risk due to stage of disease) (12). Thus, when developing an adjuvant treatment, in the absence of acute toxicity, the presented excessive relative risk per Gray (ERR/Gy), on an organ basis, should be valuable to incorporate in the recommended phase 2 activity, and it may direct the focus of optimization of the therapy to where dose reductions could be most valuable.

## 3. The present

### 3.1. Summary of the ongoing clinical trials with ^211^At

Seven ongoing clinical trials with ^211^At are summarized in Table 2. Two of these have recently opened in Japan: at Osaka University Hospital *[*^211^*At] NaAt* is being investigated in patients with differentiated thyroid cancer, and at Fukushima Medical University ^211^*At-MABG* (Meta-astatobenzylguanidine) in patients with malignant pheochromocytoma. There are five early phase clinical protocols with ^211^At-based radionuclide therapy at the Fred Hutchinson Cancer Center in Seattle, as posted on ClinicalTrials.gov (28). The common theme of the Fred Hutchinson trials is to improve outcomes after hematopoietic cell transplantation (HCT). Two approved constructs are currently under investigation, anti-CD45 (^211^*At-BC8-B10*) and anti-CD38 (^211^*At-OKT10-B10*). The rationale for these clinical trials is logical and relates to the possible ability of alpha particles to eradicate single cells and limit the dose to surrounding healthy tissues. The underlying hypothesis of these clinical trials is that the addition of highly directed cytotoxicity of ^211^At to a reduced-intensity conditioning regimen prior to HCT will reduce both late complications and early toxicity, which are frequent following high-dose systemic conditioning (29, 30). In addition to the 7 protocols described here, in Philadelphia, USA, an investigator-initiated dose-escalation trial with ^211^*At-MABG* in relapsed or primary refractory neuroblastoma is planned, which is scheduled to use the “rolling six phase I trial design” (31). Also, in Gothenburg, Sweden, we are in the end stages of concluding the necessary workup to continue i.p. treatments in ovarian cancer using a new ^211^At-construct.

**Table 2:** Ongoing and planned clinical trials with ^211^At. (NTC number) is the ClinicalTrials.gov identifier.

**Institution, reference**	**Clinical situation**	**Planned size** ** (nb Pts.)**	**Study objective(s)**	**TAT-agent/ Carrier**	**Target**	**Primary outcome**
Fred Hutchinson Cancer Center, Seattle, USA (*NCT04466475*)	Multiple Myeloma	24	Feasibility and safety	^211^At-OKT10-B10	CD38	MTD
Fred Hutchinson Cancer Center, Seattle, USA (*NCT04579523*)	Multiple Myeloma	30	Dose escalation	^211^At-OKT10-B10	CD38	MTD
Fred Hutchinson Cancer Center, Seattle, USA (*NCT04083183*)	HCT for non-malignant disease	40	Dose escalation	^211^At- BC8-B10	CD45	Graft rejection
Fred Hutchinson Cancer Center, Seattle, USA (*NCT03670966*)	High-risk acute leukemia or MDS	30	Dose-escalation	^211^At- BC8-B10	CD45	Toxicity
Fred Hutchinson Cancer Center, Seattle, USA (*NCT03128034*)	High-risk AML, ALL, MDS or Mixed-phenotype acute leukemia	50	Dose-escalation	^211^At- BC8-B10	CD45	Toxicity, MTD
Osaka University Hospital, Suita, Japan (*NCT05275946*)	Thyroid cancer	11	To establish recommended dose for Phase II trial	[^211^At] NaAt	NIS	Treatment-related adverse events
Fukushima Medical University, Japan	Malignant pheochromocytoma	Up to 18	Dose escalation	^211^At-MABG	Norepinephrine transporter	Toxicity, MTD

HCT, Hematopoietic cell transplantation.

#### 3.1.1. Seattle, USA anti-CD45

At the Fred Hutchinson Cancer Center in Seattle, translation of preclinical findings with the anti-CD45 murine IgG_1_ monoclonal construct ^211^*At-BC8-B10* has so far generated three early-phase clinical protocols that are enrolling patients (NCT03128034, NCT03670966, and NCT04083183). CD45 is expressed at high levels on the surface of all nucleated hematopoietic cells and is not internalized when bound to BC8-B10. The preclinical workup could demonstrate promising results using a canine transplantation model (32–34). Additionally, the work and data needed to obtain current good manufacturing practice (cGMP) for this radiopharmaceutical have been published (30). The NCT03128034 trial aims to evaluate escalating doses of ^211^At-labeled anti-CD45 mAb BC8 (^211^*At-BC8-B10*) followed by allogeneic HCT for high-risk acute myeloid leukemia (AML), acute lymphocytic leukemia (ALL), or myelodysplastic syndrome (MDS). It is similar in size (n=40) and the outcome measures to the NCT03670966 phase I/II trial using the same construct (^211^*At-BC8-B10*) followed by donor stem cell transplantation in the treatment of patients with relapsed or refractory high-risk acute leukemia or MDS, but differs in patient population, transplantation, and conditioning regimen.

Preliminary results were presented for the first 20 patients in the dose-escalation study with ^211^*At-BC8-B10* (NCT03128034) (35). Here, older or medically infirm adult patients with refractory/relapsed leukemia or high-risk MDS received ^211^*At-BC8-B10* i.v. for 6–8 h one week before donor HCT. The conditioning treatment included fludarabine and total body irradiation (TBI) at 2–3Gy. The MTD was defined as the primary endpoint toxicity (grade III or IV Bearman regimen-related toxicity) within the first 100 days after transplantation. The secondary endpoints include various measures of efficacy, and 50 patients can be enrolled. A single-patient dose escalation of ^211^At in increments at 1.85 MBq kg^−1^ ideal body weight was used until encountering the first dose limiting toxicity (DLT) at 20.35 MBq kg^−1^ (a bilirubin elevation), therefrom a stage 2 escalation commenced starting at 18.5 MBq kg^−1^ in cohorts of 4. The authors concluded that the preliminary efficacy data of a 1-year overall survival of 43% and recurrence-free survival of 35% support further exploration of ^211^*At-BC8-B10* in HCT for patients with high-risk AML and MDS.

The NCT04083183 phase I/II trial “Total Body Irradiation and Astatine-^211^-Labeled BC8-B10 Monoclonal Antibody for the Treatment of Non-malignant Diseases” plans to enroll 40 patients to study the best dose of total body irradiation with the ^211^*At-BC8-B10* monoclonal antibody as reduced intensity conditioning prior to HCT. This concept was addressed in a canine model of transfusion-induced sensitization and marrow graft rejection, demonstrating that the addition of ^211^At-anti-CD45 mAb to conditioning may overcome graft rejection in non-malignant diseases treated with allogeneic transplantation (36, 37). In this clinical study,^211^*At- BC8-B10* will be administered prior to induction chemotherapy (fludarabine cyclophosphamide and thymoglobulin) + TBI to patients with non-malignant diseases undergoing HCT. The primary endpoint is graft rejection, and secondary endpoints include transplant related mortality, overall survival (OS), donor chimerism, and the rate of acute and chronic graft vs. host disease (GVHD). No results from this study have yet been reported, but the two trials NCT03128034 and NCT04083183 have treated 43 patients as of July 2021 (38).

#### 3.1.2. Seattle, USA, anti-CD38

The two trials using the murine IgG_1_ anti-CD38 mAb OKT10 (NCT04466475 and NCT04579523) have similar treatment settings to the anti-CD45 trials: that is, they are aiming to treat small cell clusters or single cells, but anti-CD38 targets the malignant cells. Thus, the treatment aim is to achieve eradication of multiple myeloma minimal residual disease (MRD). The CD38 antigen is a good target expressed on malignant plasma cells, regardless of mutational status (39, 40). Cell binding and cytotoxicity from *in vitro* studies, favorable biodistribution, and *in vivo* data on efficacy using mouse models of both bulky disease and low disease burdens have been reported (41).

The NCT04466475 trial is active and recruiting. In this trial, escalating doses of ^211^*At-OKT10-B10* combined with melphalan as conditioning prior to autologous HCT in patients with multiple myeloma will be tested in 24 patients who have received at least three prior lines of therapy. The primary endpoint, MTD, is defined as a DLT probability of 25% of subjects. The secondary endpoints are response rate, duration of response, overall survival (OS), progression-free survival (PFS), and rates of MRD using flowcytometry, next generation sequencing, and functional imaging with positron emission tomography-computed tomography (PET-CT).

In the NCT04579523 trial, escalating doses of ^211^*At-OKT10-B10* followed by HLA-matched or haploidentical donor HCT for high-risk multiple myeloma will be investigated in 30 patients, assigned to one of the two arms, differing in transplant and conditioning matters. The primary endpoint is MTD. It is posted on ClinicalTrials.gov with the status of “Not yet recruiting” as of October 2022.

#### 3.1.3. Osaka, Japan, [^211^At] NaAt in thyroid cancer

Iodine is taken up by the thyroid cells by the NIS and so is astatine because of the chemical similarities, both being halogen isotopes. Currently, patients with differentiated thyroid cancer may be treated with radioactive iodine ^131^I. Research at Osaka University could demonstrate improved radiochemical purity and increased uptake of astatide in differentiated thyroid cancer cells by adding 1% ascorbic acid to the ^211^At solution, thereby stabilizing the oxidative state of ^211^At (42). Preclinical toxicity analysis (43) and a formal extended single-dose toxicity study were performed with the aim of initiating a clinical trial (44). In addition, helpful accompanying guidelines focusing on radiation safety have been published (45).

This investigator-initiated clinical trial (NCT05275946) in patients with differentiated thyroid cancer using the targeted alpha therapy drug TAH-1005 (*[*^211^*At] NaAt*) has opened for inclusion this year and so far, includes three of the 11 planned patients. This dose-escalation phase I study using a single i.v. administration of TAH-1005 is performed in patients with differentiated thyroid cancer (papillary and follicular cancer) that lack response to standard treatment. The escalating starting dose is 1.25 MBq kg^−1^, with an upper limit of 10 MBq kg^−1^. Safety, pharmacokinetics, absorbed dose, and efficacy will be evaluated to determine the recommended dose for a phase II clinical trial.

#### 3.1.4. Fukushima, Japan, ^211^At-MABG

In the mid-1990s, Meta- [^211^At] astatine-benzylguanidine (*[*^211^*At] MABG*) was shown to have superior effects to ^131^I-MIBG in the treatment of xenografted human neuroblastoma cells (46). Both of these constructs are false analogs of norepinephrine and are taken up by cells that express the norepinephrine transporter, which is also expressed in pheochromocytoma.

At Fukushima Medical University Hospital, a dose escalation phase I trial has started with ^211^*At-MABG* in patients with malignant pheochromocytoma or paraganglioma. It is based on preclinical studies, where *[*^211^*At] MABG* demonstrated therapeutic effects in malignant pheochromocytoma (47), and an investigation of acute toxicity further supported the advancement to a clinical trial (48). Also, a handling guideline for this ^211^At construct has been published (49). The study will use the 3 + 3 study design, starting with i.v. 0.65 MBq kg^−1^ and potentially escalate to 1.3 MBq kg^−1^ and 2.6 MBq kg^−1^, depending on toxicity. The primary endpoints are safety, to establish MTD, and to determine the recommended phase II dose. The secondary endpoints include pharmacokinetics, urinary radioactivity efflux rate, and measures of efficacy: urinary catecholamine response rate, overall response rate, and PFS. This study may enroll up to 18 patients.

## 4. The future

### 4.1. Clinical situations regarding the use of ^211^At

A selection of preclinical studies where ^211^At has been coupled to various vectors and their respective targets is summarized in Table 3. A few of these ^211^At-conjugates have been, or are currently being, tested in early clinical trials, as discussed in Sections 2 and 3. Most of these constructs are potential candidates for translation into clinical trials, and other vectors will surely appear. It is difficult to predict the clinical success using preclinical data. Many drug candidates with high efficacy in small-animal models have failed in humans. Therefore, rather than attempting to predict results, we show possible situations and conditions where alpha-particles and particularly ^211^At-based therapies can be of value.

**Table 3:** A selection of studies with various vectors that have been labeled with ^211^At. (IgG, immunoglobulin G).

**Malignancy**	**Target**	**TAT-agent (Vector type)**	**Ref**.
Colon cancer	Lewis Y	BR96 (IgG)	(50, 51)
Glioma	VEGFR and integrins	iRGD-C6-lys-C6-DA7R (heterodimeric peptide)	(52)
	Neurokinin receptors 1–3	Substance P (peptide)	(53)
	LAT1	Phenylalanine	(54)
	Tenascin	ch81C6 (IgG)	(15)
Head and neck cancer	CD44vs6	U36 (IgG)	(55)
Leukemia	CD20	Rituximab (IgG)	(56)
	CD45	30F11 (IgG)	(34)
	CD45	BC8 (IgG)	(33)
	CXCR4	Anti- CXCR4 (IgG)	(57)
Lymphoma	CD20	1F5 (IgG)	(58)
	CD33	Gemtuzumab (IgG)	(56)
Lung, neuroendocrine	SSTR2	Octreotide	(59)
Melanoma		Methylene blue	(60)
Multiple myeloma	CD38	OKT10 (IgG)	(41)
	CD138 (syndecan-1)	9E7.4 (IgG)	(61)
Neuroblastoma	PARP1	Parthanatine (1-(4-astatophenyl)-8,9-dihydro-2,7,9a-triazabenzo[cd]azulen-6(7H)-one)	(62)
Neuroblastoma, Pheochromocytoma	Norepinephrine transporter	Meta-benzylguanidine (MABG)	(46, 47)
Ovarian	FRα	MOv18 (IgG)	(63)
		Farletuzumab (IgG)	(64)
	NaPi2b	MX35 (F(ab')_2_)	(65)
		MX35 (IgG)	(66)
Ovarian, gastric, breast cancer	HER2	Trastuzumab (IgG)	(67, 68)
		2Rs15d (Nanobody)	(69)
		5F7 (single-domain antibody)	(70)
Various	HER2/CEA	C6.5 & T84.66 (diabodies)	(71)
Prostate	PSMA	(2S)-2-(3-(1-carboxy-5-(4-^211^At-astato-benzamido) pentyl)ureido) pentanedioic acid	(72)
	PSCA	A11 (Minibody)	(73)
	GRPR	Bombesin	(74)
Thyroid / NIS expressing tumors	NIS	Astatide	(75–77)
Various	-	Gold nanoparticles	(78)

#### 4.1.1. Personalized medicine

In recent years, the concept of precision medicine has gained increased attention owing to the development of specific drugs associated with defined genetic alterations in several tumor types. Examples include EGFR, ALK, ROS1 and RET alterations in non-small cell lung cancer, rendering tumors susceptible to tyrosine kinase inhibitors (79), or high microsatellite instability/deficient mismatch repair (MSI-H/dMMR) in gastrointestinal tumors, which is associated with response to PD1 inhibition (80). This has led to an overall belief in precision medicine as a general principle of individual patient management. Precision medicine, sometimes also called personalized medicine, primarily refers to the use of a patient's individual tumor information (e.g., genes or proteins) to guide diagnostic, treatment, or follow-up related decisions.

In radiotheranostics (81, 82), molecular imaging for diagnosis and staging, primarily PET-CT and single-photon emission computed tomography (SPECT), is combined with targeted radionuclide therapy at a later time point. It can use small molecules, peptides, or antibodies as carriers for therapeutic radionuclides, characteristically those emitting α-, β-, or auger-radiation. This radio-pharmacological personalization includes somatostatin receptor positivity in neuroendocrine tumors associated with the efficacy of ^177^Lu-DOTATATE/DOTATOC or PSMA-positive prostate cancer treated with the same radionuclide but with a different vector. The individual approach is likely to play an important role in the development of ^211^At associated treatment to increase the risk-benefit ratio and expand the treatment strategy to further tumor diagnosis or stages.

#### 4.1.2. Adjuvant therapy

Following primary therapy for a malignancy, most often surgery, small-scale disease may go undetected leading to recurrence. The risk of relapse is dependent on the type of malignancy and the disease stage at the time of treatment. This risk can be lowered with adjuvant therapy such as local post-operative external radiation therapy, pharmaceutical therapy, or endocrine therapy. Various adjuvant therapies are used for the most frequent malignancies, such as breast, colorectal, and lung cancers. Although there is a clear effect on survival, in the case of colon cancer, at most, about 30% of patients with micrometastases are cured by the chemotherapy given (83). Comparably low, or even lower, figures apply for breast and other adjuvant therapies regarding the total efficacy of adjuvant chemotherapy. Therefore, adjuvant therapy of small-scale disease using alpha-emitting radionuclides directed to malignant cells offers an appealing treatment approach because of the high LET and short path length of the alpha particles, which may prove more efficient than current standard treatments and with limited toxicity.

#### 4.1.3. Adjuvant therapy aimed on single cells

Targeted ^211^At might hold great promise as an adjuvant therapy for eradicating single cells or micrometastases remaining following primary therapies. In this setting, a much higher fraction of the radiation energy emitted from ^211^At will be deposited in the cancer cells compared to any other beta emitter. Accordingly, the tumor-to-healthy tissue ratio favors ^211^At therapy. An even better ratio has been reached for some loco-regional therapies. A good example is intraperitoneal ^211^At-radioimmunotherapy, where the calculated absorbed dose to single tumor cells and micrometastases is >20 Gy, while the bone-marrow receives <0.05 Gy (11). The low bone marrow dose was partly due to the addition of an osmotic agent that slows the transport of ^211^At-mAb from the peritoneal cavity into the circulation (20).

#### 4.1.4. Gross tumor treatment

Gross tumors, that is, macroscopic tumors, are commonly defined as tumor masses that can be detected and measured using imaging techniques, such as CT, MRI, or PET-CT. Treatment can be used for both primary and relapsed diseases. If relapse occurs at a different location from the primary site, it is referred to as metastatic disease. Until recently, metastatic disease had been considered an incurable situation for most epithelial malignancies, but this might be changing with the use of more molecular-based individual treatments. However, non-curability does not mean that a treatment is in vain. The balance between treatment-induced acute side effects and tumor effects should preferably favor low-toxicity treatments. Therapy based on ^211^At (short half-life, no serial alpha-daughters in the decay chain) may offer such treatment options.

##### 4.1.4.1. Fractionation

Diffusion and short t$\frac{1}{2}$ are arguments often used to suggest that ^211^At-based therapy might have a limited potential for success when aiming to treat larger tumor masses. Such limitations might be overcome by introducing a fractionated regime, which allows for lower cumulative bone marrow and kidney doses. This was observed in a preclinical study (84), where fractionated i.v.- radioimmunotherapy (RIT) completely eradicated small solid tumors when the cumulative tumor dose was >10 Gy. Interestingly, small-scale alpha imaging in this study revealed a markedly heterogeneous intratumoral dose-rate distribution even at relatively late time points after the injection. Pre-targeted regimens have been shown to strongly improve intratumoral diffusion and distribution of short-lived alpha-emitters at very short time points (85).

##### 4.1.4.2. PRIT

In contrast to radioimmunotherapy (RIT), pre-targeted radioimmunotherapy (PRIT) combines the ability of antibodies to target specific antigens expressed on tumor cells with the pharmacokinetic profile of a radiolabeled small molecule (effector molecule). This is used in a multistep delivery system that allows a decrease in the circulation time of radionuclides, which may reduce the dose delivered to healthy tissues. Importantly, this will facilitate the use of short-lived radionuclides that might otherwise be incompatible with antibody-based vectors (86, 87). PRIT presents added complexity in terms of dosing protocol optimization, pre-targeting intervals, and drug manufacturing. At least two products need to be developed, and perhaps a third, a clearing agent, that is needed to remove or at least reduce the unbound blood fraction from the circulation before injecting the therapeutic effector (88). Over the years, several approaches that rely on different *in vivo* ligation mechanisms have emerged. The two most studied are the non-covalent interactions of the streptavidin-biotin system and bispecific antibodies that can bind both to the tumor antigen and to a radiolabeled small molecule. Clinical investigations of both strategies have confirmed the utility of the pre-targeting approach in overcoming the high overall normal tissue radiation doses of conventional RIT (89–93) and that significant tumor doses can be achieved (87).

Other approaches include hybridization of complementary oligonucleotides and the biorthogonal inverse electron demand Diels-Alder (IEDDA) click reaction (87). Preclinical data have shown excellent potential for the clinical translation of PRIT based on the IEDDA approach (94), and clinical studies will soon be attempted (90). Complementary oligonucleotides also demonstrate high potential for application owing to some modifications to the oligomer scaffolds to prevent their *in vivo* degradation (86). Each approach has its own set of advantages and disadvantages, the challenge with PRIT lies in it being a multistep process that is difficult and costly to develop. However, PRIT has demonstrated increased value in permitting optimized reagent dosing, solving the challenge of the relatively high radiation burden on healthy tissue that has repeatedly been associated with the use of beta-emitting radioimmunoconjugates in RIT. In alpha particle-based RIT, the main benefit may lie in better tumor penetration and accompanying higher tumor doses.

### 4.2. Anatomical considerations

#### 4.2.1. Systemic treatment

Systemic treatment generally means that the drug reaches the tumor through the blood. This route of radiation delivery is needed if the malignancy initially spreads through the bloodstream. Therefore, malignancies with a high risk of liver, lung, or bone marrow metastasis are likely to be well-suited for systemic delivery. Logistically, it is a good administration route because of the ease of access; however, when the activity is at its highest, all normal organs are exposed to unspecific irradiation in proportion to the organ blood flow. This drawback is more pronounced when radionuclides with shorter t$\frac{1}{2}$ values are used.

#### 4.2.2. Intra cavitary treatment

The first two clinical studies used an intra cavitary treatment situation (8, 9). By doing so, normal organ exposure can be significantly reduced, which increases the therapeutic window. This is the logical choice if the main clinical problem is local regrowth or relapse.

##### 4.2.2.1. Abdominal cavity – i.p. treatment

Ovarian cancer is an archetypical malignancy with a high rate of intraperitoneally relapses, even after successful surgery and chemotherapy. In fact, ~70–80% of patients with epithelial ovarian cancer will develop disease relapse (95). However, in gastric, colorectal, and pancreatic cancers, i.p. directed therapy can be useful to reduce local recurrences and associated morbidity. High rates of peritoneal recurrence are for example common following gastric cancer surgery, ranging from 35 to 60% (96). In colon cancer, the incidence of peritoneal metastases during follow-up has been estimated to be 70–80% if positive resection margins or peritoneal nodules are detected during surgery (97, 98). Moreover, pancreatic cancer has a high risk of eventually developing peritoneal metastases, with ~10% in first recurrences but up to 40–60% in advanced stages (99). Thus, the clinical trial in ovarian cancer with ^211^At based radioimmunotherapy (section 2.1.4) may be followed by trials in other clinical malignancies using a similar treatment set-up.

##### 4.2.2.2. Fluid evacuation

Preclinical studies of i.p.-RIT have shown that an improved therapeutic window could be achieved with an accelerated post-administration fluid evacuation and performing peritoneal flushing (100, 101). Using this strategy, the normal tissue organ uptakes was significantly decreased, while the tumor uptakes was preserved (100). This corresponded to an increase in the tumor-to-normal-tissue mean absorbed dose-rate ratio (TND) for blood from 1.7 to 6. This concept was also evaluated in a study using the short-lived alpha-emitter ^213^Bi, where the TND for blood increased from 1.3 to 6 (101).

##### 4.2.2.3. Spinal canal, ventricular system -intrathecal treatment

Besides the intra cavitary treatment used in the first ^211^At clinical trial (section 2.1.3), other central nervous system (CNS)-located diseases such as neuroblastoma or leptomeningeal metastases have been treated with radioimmunotherapy to achieve better control of minimal residual disease. Intrathecal targeted radiation was introduced at the Memorial Sloan-Kettering Cancer Centre (MSKCC) in New York. Clinical studies have so far involved electron-emitter ^131^I conjugated to murine 3F8 (anti-GD2) or 8H9 (anti-B7H3) antibodies (102–104). To this end, the MSKCC team has published several pharmacokinetic models of intrathecal RIT (105–108). They also modeled alpha-emitter ^225^Ac and stated that “as new novel radioisotopes and their microdosimetry become available, further improvement in the pharmacokinetic modeling of CNS-RIT modality should refine this emerging therapy to fit the clinical context” (105). Indeed, recently presented pharmacokinetic models and calculated microdosimetry for intrathecal administered ^211^At-labeled 3F8 and 8H9 antibodies are promising (109).

##### 4.2.2.4. Other intra cavitary treatments

Local therapy is, as shown above, an attractive and feasible treatment option. Therefore, in addition to the discussed intraperitoneal and intrathecal body cavities, local treatment may be envisioned in the pleural space following, for example, surgery for mesothelioma, or in palliative care to reduce malignant effusions in the abdomen or pleural cavity by using an appropriate vector with successful stable ^211^At chemistry.

### 4.3. Modeling to enhance the therapeutic window

Models of ^211^At-radioligand binding and retention to cancer cells (110) combined with microdosimetry (111) and biokinetic models (20) have generated proposals that may optimize radionuclide therapies in the above-mentioned clinical situations. Examples include the use of an osmotic agent in intraperitoneal radioimmunotherapy, mainly to reduce bone marrow absorbed doses (20). Another suggestion from modeling is to add a “cold,” i.e., non-radiolabeled, antibody as a post-therapy boost aiming at increasing the absorbed dose to the core of slightly larger microtumors (110).

### 4.4. Vectors and radiolabeling with ^211^At

#### 4.4.1. The chemistry

Most alpha-emitting radionuclides are radiometals, for which metal chelators can be used for radiolabeling targeting vectors, whereas ^211^At is a radio-halogen. Generally, halogen properties can be applied in astatine labeling chemistry, but in contrast to iodine chemistry, they cannot be applied in the direct labeling of proteins (112). It was found very early on that both the chemistry and *in vivo* behavior of astatine were different from those of iodine (113, 114), the closest neighbor in the halogen group. Although astatine is a halogen, it also has metallic properties. However, no efficient chelator has been developed for astatine. The chemistry of astatine has been difficult to fully elucidate, to which its low availability has contributed negatively. However, with the increased interest in its use in TAT, much effort has been made to understand its properties in recent years (5). In principle, two main types of bonds are used for astatine labeling: covalent bonds to aromatic groups and binding to boron cages (115, 116). Several different methods for covalent bonding of astatine have been developed such as the use of boronic acid leaving groups and iodonium salts; however, the most commonly used and well-established method is electrophilic destannylation of an aryl organo-tin group (5, 117). For the radiolabeling of proteins and other vectors, the most common approach is the use of an intermediate bifunctional reagent that includes an amino directing group for conjugation to the vector, for example, an aryl organo-tin group for labeling with ^211^At (118). The issue of *in vivo* stability is strongly connected to the radiochemistry methodologies used with astatine. A number of animal studies using ^211^At have observed that uptake in normal organs, such as the stomach, spleen, and lungs, was elevated. In most cases, this is likely due to *in vivo* deastatination. Much effort has been put into improving the radiolabeling methods for ^211^At (5).

#### 4.4.2. Vectors

A wide range of vector types have been radiolabeled with ^211^At. Table 3 provides a non-comprehensive summary of these examples. Antibodies have been one of the main vectors for guiding ^211^At to the tumor site, and basically all types of antibodies can be astatinated using the intermediate reagents discussed above. However, although alpha-radioimmunotherapy with ^211^At is well-suited for local compartment applications such as intracavitary or intraperitoneal treatments, general treatments using a systemic administration route (generally i.v.) are limited by a slow distribution to the tumor tissue and the clearance rate of antibodies, resulting in slow accumulation in the tumor. To circumvent the unfavorable pharmacokinetics of radiolabeled antibodies, pre-targeting techniques (see above section on PRIT) can be employed. In addition, pharmacokinetics can be optimized utilizing smaller protein vectors, such as nanobodies or minibodies, to better match the half-life of ^211^At. In addition, small astatinated organic molecules, such as phenylalanine and MABG (Table 3), display a significantly faster distribution pattern than antibodies. With both these types of constructs, one must take clearance through the kidneys into account to avoid nephrotoxicity.

### 4.5. Treatment availability

#### 4.5.1. Nuclide production

Astatine is one of the rarest elements on Earth; therefore, ^211^At must be artificially produced. The main production route today is to irradiate a bismuth-209 target in a cyclotron capable of producing a 28 MeV alpha beam. The alpha beam transforms the target bismuth into ^211^At by the nuclear reaction ^209^Bi(α,2n)^211^At (119). Astatine-211 can also be produced by heavy-ion irradiation of bismuth using the nuclear reaction ^209^Bi(^7^Li,5n)^211^Rn and subsequently using^211^Rn as a generator of ^211^At (3, 120). Isolation of astatine from the spallation reaction is also possible. Comparing the production routes, ^209^Bi(α,2n)^211^At is the most straight forward and is likely to be the main route to prevail (3, 4). Currently there are 13 cyclotron facilities that produce ^211^At (3). However, several efforts have been made to increase the capacity to meet the demand of ^211^At. Approximately 30 production sites are or will shortly be available. Currently, three manufacturers are producing medium-energy cyclotrons with the capacity of an alpha beam. The Ion Beam Applications (IBA) in Belgium, has the multi-particle machine Cyclone 30XP in stock. Sumitomo (Japan) produce the MP-30 cyclotron. Although not yet on the market, Ionetix (USA), is developing new mono-energetic machines for ^211^At production. In addition to cyclotron production, linac production has also attracted attention (121). Linac machines can apply a very high current to the target and potentially produce high amounts of ^211^At. However, the main hurdles to overcome with linac production are few facilities (i.e., beam time) and targetry.

#### 4.5.2. Logistics

The logistics of this type of treatment, utilizing a relatively short-lived nuclide, concern several factors that carry different importance depending on geographical location and national nuclear medicine healthcare traditions. Various local logistical concerns may include the produced nuclide itself, either as a target or a purified fraction, the radiolabeled precursor, the completely synthesized radiopharmaceutical, the patient to be treated, or a combination of these. This creates a complex system where no single solution fits all, as recently reviewed (3, 4). Importantly, the clinical trial performed in Gothenburg, Sweden, received the ^211^At from the cyclotron at Rigshospitalet, in Copenhagen, Denmark, proving that a production site can be situated up to approximately 3 h away from the where the radiopharmacy and treatment takes place (8). However, for routine clinical treatment with an astatine-containing radiopharmaceutical, there is a need for automatic recovery of the produced nuclide from the solid target as well as the subsequent radiopharmaceutical synthesis. Several research groups have identified this need, and efforts have been made to automate nuclide recovery with wet extraction (122), solid-phase extraction (123), and dry distillation in combination with radiopharmaceutical synthesis (124).

#### 4.5.3. ***Collaborative initiative***s

In Europe, the EU-funded project Network for Optimized Astatine labeled Radiopharmaceuticals (NOAR) was started in 2020, supported by the funding organization of the European Cooperation in Science and Technology (COST). NOAR has addressed the specific question of the future logistics of astatine-based radiopharmaceuticals in terms of production capacity, recovery processes, and transnational movement of patients to specific treatment nodes (125). In the United States, the U.S. Department of Energy (DOE) has a specific isotope program where the National Isotope Development Centre is set out to “support the US Department of Energy Isotope Program as the global leader in the production and distribution of radioactive and enriched stable isotopes that are deemed critical or are in short supply,” and where one of the nuclides in focus is ^211^At (126). In Japan ^211^At based research has been very efficacious, and two clinical trials have been initiated within a short period of time. Part of this success is due to the creation of a nationwide supply chain from five ^211^At production facilities to more than 18 end-user facilities.

### 4.6. Summary

Only two clinical trials have been performed to date, but presently, seven different protocols are underway, and two more may be starting within a short period. To date, all performed and scheduled trials are small safety or dose-finding trials, and none have a control population. Hopefully, larger effect-seeking studies, preferably randomized studies, will likely start within a few years once the recommended phase 2 dose has been set. Collaborative initiatives that have started in Europe, Japan, and the USA will facilitate and focus on ongoing research. If joined, these collaborations could clearly aid in the launch of international multicenter controlled clinical trials with ^211^At-based radiopharmaceuticals.

## Author contributions

PA, TB, and SP contributed to the design, concept, and completion of this article. All the authors listed contributed with substantial, direct, and intellectual contribution to the work and approved the submitted version for publication.
